# Diagnostic Performance and Misclassification Patterns of Preoperative MRI in Rectal Cancer: A Real-World Study

**DOI:** 10.3390/diagnostics16101481

**Published:** 2026-05-13

**Authors:** David Luengo Gómez, Ángel Francisco Ávila Jiménez, Miguel Ángel Araújo-Jiménez, Encarnación González Flores, Consolación Melguizo Alonso, Mercedes Zurita Herrera, Antonio Jesús Láinez Ramos-Bossini, Ángela Salmerón Ruiz

**Affiliations:** 1 Department of Radiology, Hospital Universitario Virgen de las Nieves, 18014 Granada, Spain; david.luengo.sspa@juntadeandalucia.es (D.L.G.); angela.salmeron.sspa@juntadeandalucia.es (Á.S.R.); 2 Instituto de Investigación Biosanitaria de Granada (ibs.GRANADA), 18012 Granada, Spain; encarnacion.gonzalez.flores.sspa@juntadeandalucia.es (E.G.F.); melguizo@ugr.es (C.M.A.); mercedes.zurita.sspa@juntadeandalucia.es (M.Z.H.); 3 Department of Human Anatomy and Embryology, Faculty of Medicine, University of Granada, 18016 Granada, Spain; angelfranaj@gmail.com (Á.F.Á.J.); mikelanchelo000@gmail.com (M.Á.A.-J.); 4 Department of Medical Oncology, Hospital Universitario Virgen de las Nieves, 18014 Granada, Spain; 5 CIBER de Epidemiología y Salud Pública (CIBERESP), 28029 Madrid, Spain; 6 Institute of Biopathology and Regenerative Medicine (IBIMER), University of Granada, 18100 Granada, Spain; 7 Center of Biomedical Research (CIBM), University of Granada, 18100 Granada, Spain; 8 Department of Oncological Radiotherapy, Hospital Universitario Virgen de las Nieves, 18014 Granada, Spain

**Keywords:** rectal cancer, magnetic resonance imaging, staging, restaging, diagnostic accuracy

## Abstract

**Introduction**: Magnetic resonance imaging (MRI) is the reference imaging modality for locoregional staging and restaging of rectal cancer (RC). However, its agreement with surgical pathology in real-world practice is limited. We aimed to assess the agreement and diagnostic performance of preoperative MRI for dichotomized T and N staging in RC. Secondarily, we explored the direction of MRI misclassification and potential preoperative factors associated with discordance. **Methods**: We conducted a retrospective real-world study on 152 consecutive patients with pathologically confirmed RC who underwent surgery between September 2019 and June 2025 in our institution. Two cohorts were analyzed separately: patients treated without neoadjuvant therapy (non-NAT, n = 70) and patients treated with NAT followed by restaging MRI and surgery (NAT, n = 82). The main staging outcomes were dichotomized into T0-T2 vs. ≥T3 and N0 vs. N+, using final pathology as the reference standard. Agreement, Cohen’s kappa, sensitivity, specificity, predictive values, McNemar’s test, and exploratory regression analyses for misclassification were performed. **Results**: In the overall cohort, agreement was 72.4% for T staging and 73.0% for N staging, with moderate agreement for T (kappa = 0.452) and fair-to-moderate agreement for N (kappa = 0.349). Sensitivity and specificity were 80.3% and 67.0% for T staging and 54.5% and 80.6% for N staging, respectively. T-stage errors were mainly associated with overstaging. In NAT-treated patients, baseline MRI showed markedly poorer agreement with final pathology than restaging MRI, particularly for T stage (45.1% vs. 72.0%). Exploratory analyses did not identify strong or reproducible predictors of misclassification. **Conclusions**: This real-world study provides a contemporary estimate of MRI-pathology agreement for dichotomized T and N staging in routine RC care. Agreement was moderate, and performance was more consistent for advanced T-category assessment than for nodal staging. These findings support MRI as a practical tool for multidisciplinary risk stratification and highlight the need for continued monitoring of MRI usage and performance in clinical practice.

## 1. Introduction

Colorectal cancer (CRC) represents the third most common type of cancer (9.6%) and the second leading cause of cancer-related death (9.3%) worldwide [1], with rectal cancer (RC) accounting for approximately one-third of all cases. The incidence of RC has experienced a significant increase in recent years in young adults (<50 years old), who are expected to account for 25% cases of all RC cases by 2030 [2]. Recent advances such as Total Neoadjuvant Therapy (TNT) and Watch and Wait strategies are rapidly changing the state-of-the-art in the management of RC [3,4], and they heavily rely on accurate diagnosis and risk stratification, for which multidisciplinary collaboration is essential.

In this context, MRI has become a major advance in local staging of RC due to its excellent soft tissue resolution and ability to provide multiplanar images, allowing precise anatomical classification of rectal tumors and assessment of several prognostic factors, such as the tumor’s relationship to the mesorectal fascia (MRF), anterior peritoneal reflection, or sphincter complex [5]. Currently, MRI is a cornerstone for staging and risk stratification according to the TNM system [6] and, in the context of state-of-the-art neoadjuvant treatment (NAT) approaches, it has also become essential in RC restaging [7]. In the latter context, particularly when organ-preservation strategies are being considered, reassessment is based on a multimodal evaluation (combining MRI, endoscopy, and digital rectal examination) to estimate the degree of response and to classify patients as having complete, near-complete, or incomplete clinical response [8].

Despite being the gold standard for non-invasive staging and restaging of RC, MRI still faces several challenges, such as limited accuracy in distinguishing T1 from T2 stage, fibrotic tissue from residual tumor, or neoplastic involvement of small lymph nodes [9]. Several causes may lead to misinterpretation of MRI by radiologists, reducing the correlation between radiological and histopathological findings. For instance, following NAT, it is usually difficult to distinguish between mesorectal fat involvement and desmoplastic or fibrotic reaction, which may result in T-category overstaging [10]. Other common pitfalls on MRI include actinic and inflammatory changes, asymmetric wall collapse, pseudolesions on the external surface, extracellular mucin, or imaging artifacts [11]. Importantly, histopathology based on TNM classification remains the gold standard for definitive RC local staging [8,12].

Previous literature shows great heterogeneity in the diagnostic performance of MRI for RC staging and restaging, contingent upon a number of factors such as the endpoint assessed, clinical setting, or reader expertise [5,13]. This heterogeneity is particularly relevant for T and N categorization, whereas performance is more consistent for dichotomized outcomes and high-risk features such as MRF involvement [14]. In general, MRI tends to overstage disease compared with pathology [15], particularly after NAT. In this context, recent consensus guidelines have emphasized the importance of structured MRI interpretation and reporting, as well as the need to focus not only on anatomical TNM categories but also on clinically relevant risk stratification and, after NAT, response-oriented reassessment [7,8].

Although MRI is firmly established as the reference imaging modality for local staging and restaging of RC, its real-world performance remains dependent on the clinical context, reader interpretation, and reporting quality [16]. In this rapidly evolving setting, real-world studies with pathological correlation help assess and monitor the performance of MRI in routine clinical practice. Such studies are particularly relevant because they evaluate the final imaging classification used for multidisciplinary decision-making, rather than isolated reader performance under experimental conditions. Accordingly, the aim of this study was to assess the agreement and diagnostic performance of preoperative MRI compared with surgical pathology for dichotomized T and N staging in RC. Secondarily, we explored the direction of MRI misclassification and potential preoperative factors associated with discrepancies between MRI and pathology.

## 2. Materials and Methods

### 2.1. Study Design and Eligibility Criteria

We conducted a retrospective observational real-world study based on a consecutive series of patients diagnosed with RC between September 2019 and June 2025 at Hospital Universitario Virgen de las Nieves (Granada, Spain), a tertiary referral center attending a population of approximately 500,000 inhabitants. The study was approved by the Provincial Ethics Committee of Granada (RECRAD25; approval date: 28 October 2025). Given the retrospective nature of the study, the requirement for written informed consent was waived. The design and reporting of the study followed the recommendations of the STROBE (Strengthening the Reporting of Observational Studies in Epidemiology) statement [17].

All cases were retrospectively identified from the records of the Multidisciplinary Colorectal Cancer Committee. Eligible patients were consecutive adults (>18 years) with histopathologically confirmed rectal adenocarcinoma who underwent surgery and had an available preoperative rectal MRI examination. The exclusion criteria were as follows: (1) unavailable MRI studies or non-diagnostic MRI examinations due to artifacts; (2) absence of complete tumor resection with corresponding histopathological diagnosis of the surgical specimen; and (3) inability to complete the planned treatment regimen in the NAT group.

Two clinical cohorts were analyzed separately. In the first cohort, patients underwent surgery after initial MRI staging, without NAT. Patients in the NAT cohort received NAT according to multidisciplinary decision-making, including chemotherapy and/or radiotherapy-based regimens, followed by restaging MRI and surgery. The MRI examination considered for the main analysis was defined according to the therapeutic pathway: baseline staging MRI in patients who proceeded directly to surgery without NAT, and restaging MRI in patients treated with NAT before surgery.

No formal a priori sample size calculation was performed due to the real-world feasibility-based design, and all consecutive eligible patients within the study period were included. All MRI examinations were acquired on 1.5 T and 3.0 T scanners (SIGNA Artist and SIGNA Architect, respectively; GE Healthcare, Waukesha, WI, USA)using internationally validated rectal MRI protocols, as described in [18,19]. All MRI examinations were initially reported by an experienced abdominal radiologist from a pool of six readers and subsequently reviewed by a second radiologist before multidisciplinary discussion. Thus, all studies underwent double reading, and the final MRI classification used in the present study was the one adopted for the multidisciplinary tumor board. Histopathological assessment of the surgical specimens was performed by expert gastrointestinal pathologists according to international recommendations.

### 2.2. Study Variables

For both cohorts, the following variables were collected: sociodemographic variables (age and sex); treatment-related variables (e.g., treatment pathway and neoadjuvant regimen); and tumor-related radiological and histopathological variables, including anatomical location within the rectum, mucinous component, MRF involvement, extramural vascular invasion (EMVI), invasion of the peritoneal reflection, and tumor deposits, among others.

The main dependent variables were the T and N categories, dichotomized as T0-T2 vs. ≥T3 and N0 vs. N+, respectively, as in previous studies [20,21,22]. This dichotomization was grounded on the clinically relevant distinction between so-called early RC (e.g., cT1-2, N0), usually managed with surgery, and locally advanced RC (e.g., cT3-4 and/or N+), which is commonly managed with NAT [5]. The reference standard was the final histopathological staging of the surgical specimen.

For the main agreement analyses, MRI findings were compared with pathology using the baseline MRI in the non-NAT cohort and restaging MRI in the NAT cohort. Secondarily, we also explored the agreement between baseline staging MRI findings and pathology in the NAT cohort. In addition to dichotomized T and N staging, binary variables were created to define MRI misclassification for each endpoint, i.e., disagreement between MRI and pathology for dichotomized T stage and for dichotomized N stage. The direction of discrepancy was also classified as overstaging or understaging.

### 2.3. Statistical Analysis

First, descriptive analyses were performed for the overall cohort and separately for the non-NAT and NAT cohorts. Categorical variables were summarized using absolute and relative frequencies. Quantitative variables were summarized as mean and standard deviation (SD) when normally distributed, or as median and interquartile range (IQR) otherwise. Distributional assumptions were assessed using the Shapiro–Wilk test.

Second, an agreement and diagnostic performance analysis was performed for dichotomized T and N staging. For each endpoint, contingency tables were constructed and the following measures were estimated: overall agreement, Cohen’s kappa coefficient, sensitivity, specificity, positive predictive value (PPV), and negative predictive value (NPV). Exact binomial 95% confidence intervals (95% CI) were calculated for overall agreement, sensitivity, specificity, positive predictive value, and negative predictive value. Confidence intervals for Cohen’s kappa were calculated using standard asymptotic methods. McNemar’s test was also used to explore systematic differences between MRI-based and pathology-based classification. Agreement analyses were performed in the overall cohort and stratified by treatment pathway (non-NAT and NAT). In the NAT cohort, an additional supplementary agreement analysis comparing baseline MRI with final pathology was also performed.

Third, bivariable analyses were performed to explore factors potentially associated with MRI classification error. These analyses were conducted according to the presence or absence of incorrect classification for dichotomized T and N categories. Additional exploratory analyses distinguishing overstaging from understaging were also performed. Categorical variables were compared using the chi-square test or Fisher’s exact test, as appropriate. Quantitative variables were compared using Student’s *t*-test or the Mann–Whitney U test, depending on distributional assumptions.

Finally, univariable and multivariable logistic regression analyses were performed using preoperative variables to identify potential predictors of MRI misclassification for T and N staging. Separate models were fitted for T misclassification and N misclassification. Multivariable analyses were then performed using a parsimonious strategy due to the expected limited number of discordant cases. Specifically, penalized logistic regression with Firth’s correction was used to reduce small-sample bias and instability, and predictors with a univariable *p*-value < 0.15 were considered for inclusion, including cohort into pooled models.

Primary analyses were based on complete cases for each endpoint. All analyses were performed using R software (version 4.5.0). All tests were two-sided, and *p* < 0.05 was considered statistically significant.

## 3. Results

### 3.1. Study Population and Baseline Cohort Characteristics

**Figure 1:**
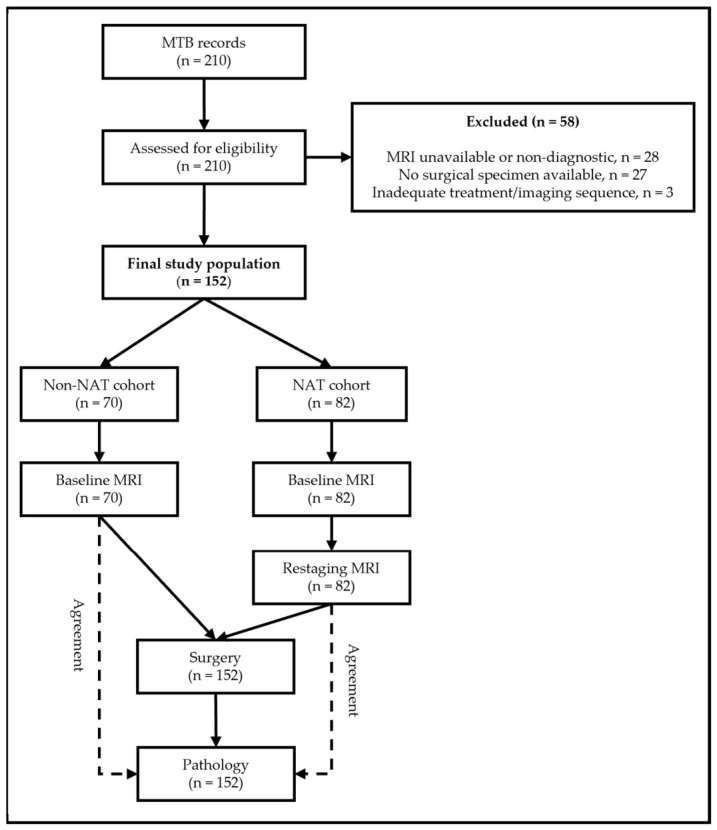
Study flow diagram. Flowchart of patient selection from the colorectal cancer multidisciplinary board records to the final non-NAT and NAT cohorts. Note that a supplementary baseline MRI agreement analysis was also conducted within the NAT cohort. MTB, multidisciplinary tumor board.

From an initial population of 210 candidates, following the inclusion and exclusion criteria, a total of 152 consecutive eligible patients were included in the study, comprising 70 patients in the non-NAT cohort and 82 in the NAT cohort. The flow diagram of the study is shown in Figure 1.

Patients in the NAT cohort were slightly younger than in the non-NAT cohort (64.34 ± 10.74 vs. 68.06 ± 9.17 years, *p* = 0.023), whereas sex distribution was similar between groups (male sex, 62.2% vs. 64.3%; *p* = 0.867). Tumor location also differed significantly between cohorts (*p* = 0.001), with a higher proportion of upper rectal tumors in the non-NAT cohort and more mid/upper or lower/mid tumors in the NAT cohort.

Baseline MRI findings indicated a more advanced radiological profile in the NAT cohort. Compared with the non-NAT cohort, NAT patients showed greater tumor thickness on baseline MRI (median 13.25 mm vs. 8.75 mm; *p* < 0.001), more frequent MRF involvement (32.9% vs. 1.4%; *p* < 0.001), more frequent EMVI (29.3% vs. 7.1%; *p* < 0.001), more tumor deposits (7.3% vs. 0%; *p* = 0.031), and more metastatic disease at baseline MRI (15.9% vs. 2.9%, *p* = 0.012).

Similarly, the distribution of T and N categories on baseline MRI differed significantly between cohorts (*p* < 0.001). However, these between-cohort differences were less pronounced in the preoperative (i.e., post-NAT) MRI used for the main analysis, with no significant difference in main MRI N stage (*p* = 0.650) and borderline differences in main MRI T stage (*p* = 0.054). Finally, pathological T and N stage distributions did not differ significantly between cohorts (*p* = 0.455 and *p* = 0.326, respectively).

Of note, a small number of patients in the non-NAT cohort had adverse baseline MRI features such as EMVI or peritoneal reflection invasion. These patients were maintained in the non-NAT cohort because treatment pathway classification was based on the actual multidisciplinary decision and management received, rather than on individual MRI features in isolation. Baseline characteristics of the sample are summarized in Table 1, and the full description is provided in Supplementary Table S1. In addition, treatment-related variables in the NAT cohort can be consulted in Supplementary Table S2.

### 3.2. Agreement and Diagnostic Performance of Preoperative MRI

In the overall cohort, MRI achieved an agreement of 72.4% for dichotomized T staging and 73.0% for dichotomized N staging. Agreement was moderate for T staging (kappa = 0.452) and fair-to-moderate for N staging (kappa = 0.349). For T staging, sensitivity was 80.3%, specificity 67.0%, PPV 62.0%, and NPV 83.6%. For N staging, sensitivity was lower at 54.5%, whereas specificity remained higher at 80.6%; PPV and NPV were 53.3% and 81.3%, respectively. McNemar’s test suggested significant directional disagreement for T staging in the overall cohort (*p* = 0.009), but not for N staging (*p* = 1.000).

In the non-NAT cohort, agreement was 72.9% for T staging and 68.6% for N staging. MRI showed high sensitivity for detecting pathologically advanced T stage (88.5%) but lower specificity (63.6%). Conversely, performance for N staging was weaker, with a sensitivity of 44.4% and specificity of 76.9%. Directional disagreement remained significant for T staging (*p* = 0.006), but not for N staging (*p* = 0.831).

In the NAT cohort, agreement was 72.0% for T staging and 76.8% for N staging. For T staging, sensitivity and specificity were 74.3% and 70.2%, respectively. For N staging, sensitivity was 61.5% and specificity 83.9%. In contrast to the non-NAT cohort, McNemar’s test did not indicate significant directional disagreement for either T or N staging in the NAT cohort (*p* = 0.404 and *p* = 1.000, respectively).

**Figure 2:**
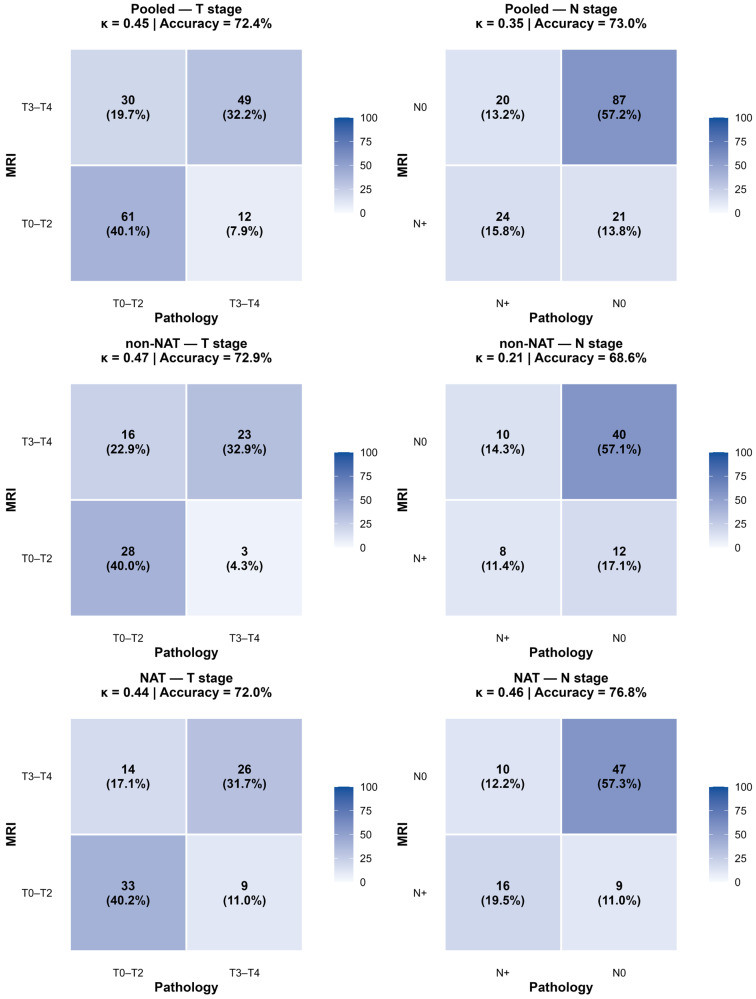
Concordance heatmaps for dichotomized MRI versus pathological staging. Heatmaps show the distribution of concordant and discordant classifications between MRI and surgical pathology for dichotomized T and N stages in the pooled cohort, the non-NAT cohort, and the NAT cohort.

Agreement and diagnostic performance metrics for dichotomized T and N staging are shown in Table 2 and Supplementary Figure S2, with the corresponding heatmaps in Figure 2.

### 3.3. Direction of MRI Misclassification

**Figure 3:**
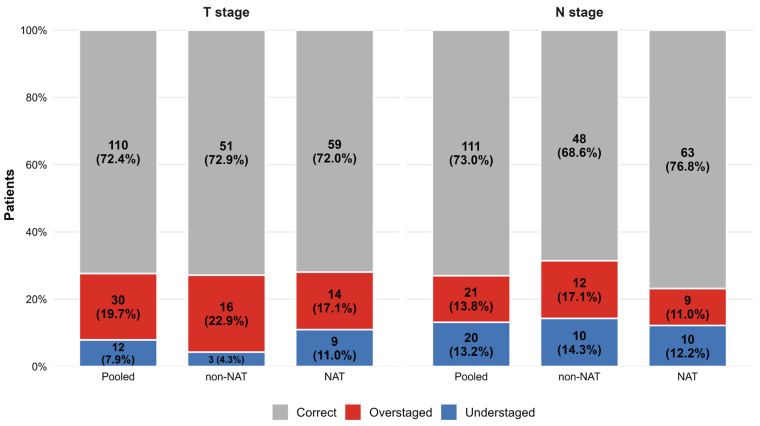
Direction of MRI misclassification for dichotomized T and N staging. Stacked bar plots show the proportions of correct classification, overstaging, and understaging for dichotomized T and N categories, using surgical pathology as the reference standard. Results are shown for the pooled cohort, the non-NAT cohort, and the NAT cohort. Percentages were calculated within each subgroup and endpoint. Note that minor deviations from 100% are due to rounding to one decimal place.

Overall, 110 of 152 patients (72.4%) were correctly classified for dichotomized T stage and 111 of 152 (73.0%) for dichotomized N stage. The confusion matrices are shown in Supplementary Table S3, and the direction of misclassification is summarized in Figure 3.

**Figure 4:**
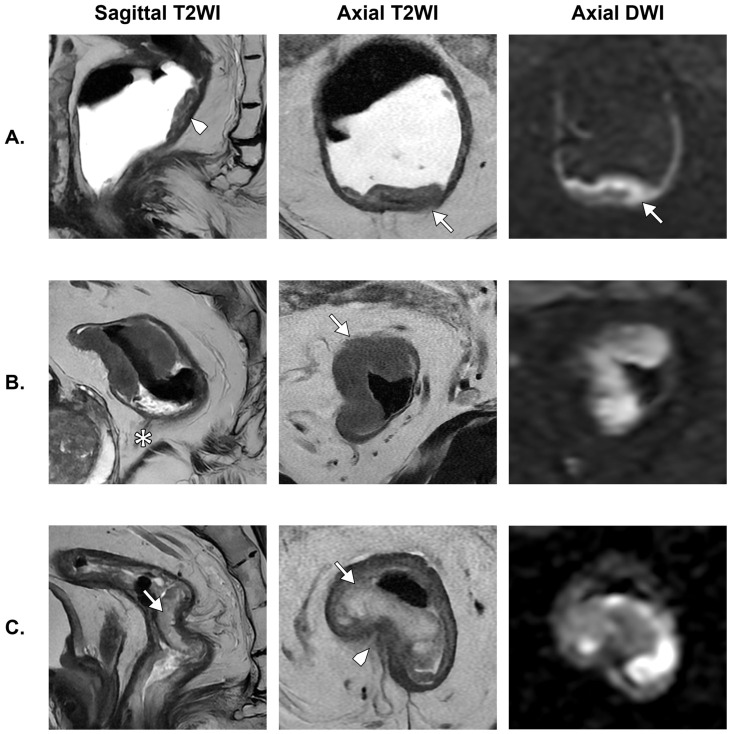
Examples of T-stage misclassification in the non-NAT cohort. (**A**) A male patient with a small posterior mid-rectal tumor (arrowhead). High-resolution T2WI suggests confinement to the muscularis propria, with subtle focal extramural extension on T2WI and DWI (arrows), leading to overstaging as T3a. Histopathology demonstrated a T1 tumor. (**B**) A male patient with a semicircumferential upper rectal tumor located above the anterior peritoneal reflection (asterisk). Axial T2WI shows apparent anterior extension into the mesorectal fat (arrow), suggestive of T3b disease. Histopathology confirmed a T2 tumor. In both cases, overstaging is likely related to partial volume effect. (**C**) A male patient with mucinous mid-rectal adenocarcinoma arising from a tubulovillous adenoma. Marked intrinsic T2 hyperintensity led to underestimation of tumor extent, with emphasis on hyperintense areas (arrows) and under-recognition of intermediate T2 signal (arrowhead). The lesion was initially classified as T1–T2; however, histopathology demonstrated extramural invasion (T3).

For T staging in the overall cohort, misclassification was predominantly related to overstaging rather than understaging. Specifically, 30 patients were overstaged and 12 were understaged relative to pathological T category. This pattern was most pronounced in the non-NAT cohort, in which 16 patients were overstaged and only 3 were understaged. In the NAT cohort, the discrepancy was less marked, with 14 overstaged and 9 understaged cases. Figure 4 shows illustrative examples of T misclassification errors on MRI.

**Figure 5:**
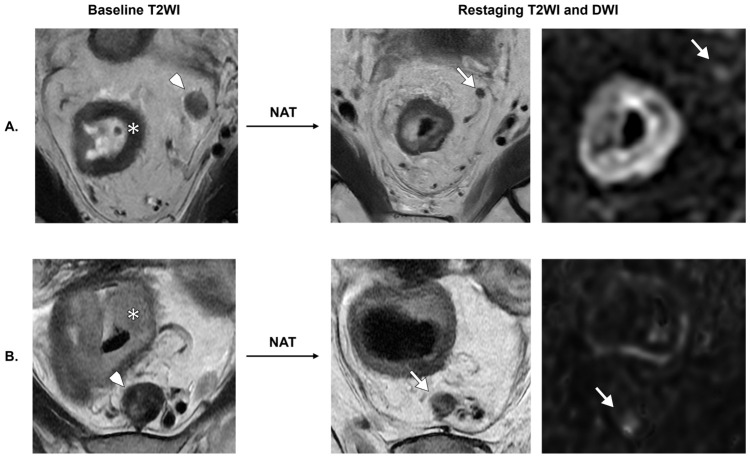
Two examples of nodal staging errors in patients with rectal cancer treated with NAT. (**A**) A female patient with locally advanced rectal cancer (asterisk). Baseline MRI showed mesorectal nodal disease, including a prominent lateral mesorectal node (arrowhead). After total neoadjuvant therapy, only a small residual node <5 mm was identified at the same location (arrows), suggesting complete nodal response. Histopathology revealed 16 lymph nodes, 3 of which contained residual tumor (partial response), consistent with N1b disease. (**B**) A female patient with a mid-rectal tumor (asterisk) and a large posterior mesorectal node at baseline (arrowhead). After chemoradiotherapy, the node decreased in size (arrows) but remained >5 mm in short axis, leading to classification as N1a. Histopathology identified 13 lymph nodes, all negative for viable tumor, showing mucinous degeneration without associated neoplastic cells.

In contrast, N-stage misclassification was more balanced in direction. In the overall cohort, 21 patients were overstaged and 20 were understaged. A similar balance was observed within each treatment subgroup: 12 overstaged versus 10 understaged cases in the non-NAT cohort, and 9 versus 10 in the NAT cohort. This symmetric error pattern is concordant with the non-significant McNemar tests for N staging across all main analyses. Figure 5 shows illustrative examples of N misclassification errors on MRI.

### 3.4. Comparison of Baseline MRI with Pathology in the NAT Cohort

The secondary analysis comparing baseline MRI with pathology (Table 3) showed markedly lower agreement than the main NAT restaging analysis, particularly for T staging. Agreement between baseline MRI and pathology was 45.1% for dichotomized T stage (kappa = 0.037) and 52.4% for dichotomized N stage (kappa = 0.154).

For T staging, baseline MRI in NAT showed very high sensitivity but extremely poor specificity when compared with final pathology. The corresponding confusion matrix showed 45 overstaged cases and no understaged cases for T stage. For N staging, baseline MRI also tended to overstage disease, with 34 overstaged and 5 understaged cases. McNemar’s test confirmed strong directional disagreement for both T and N (both *p* < 0.001).

### 3.5. Exploratory Analyses of Factors Associated with MRI Misclassification

Overall, no consistent predictors of T-stage misclassification were observed. In the pooled cohort, none of the evaluated baseline MRI variables showed a statistically significant association with T misclassification in bivariable analyses. In the NAT cohort, younger age was associated with T misclassification in the bivariable analysis, remaining borderline in exploratory regression models. However, no stable multivariable model could be derived for T misclassification in the non-NAT cohort because of sparse data and limited informativeness.

For N-stage misclassification, the strongest overall signal was observed for peritoneal reflection invasion on baseline MRI. In the pooled bivariable analysis, this feature was more frequent among misclassified cases. In exploratory regression analyses, tumor location also showed an association with N misclassification in the pooled cohort, whilst, in cohort-specific models, age and peritoneal reflection invasion showed borderline associations in the NAT cohort.

Exploratory bivariable analyses according to pathological T and N stage are presented in Supplementary Tables S4 and S5, whereas analyses of MRI misclassification are shown in Supplementary Tables S6–S10.

## 4. Discussion

In this real-world retrospective study, we provide a contemporary estimate of the diagnostic performance of preoperative MRI compared with surgical pathology for dichotomized T and N staging in RC. Overall, MRI showed moderate agreement with surgical pathology. Although overall agreement was similar for both outcomes, MRI performed more favorably for T than for N staging, with higher sensitivity for advanced T category and a clearer directional pattern of error. Specifically, T-stage discrepancies were predominantly related to overstaging, whereas N-stage misclassification was more balanced between overstaging and understaging. In addition, the secondary comparison between baseline MRI and final pathology in NAT-treated patients showed a significant decrease in agreement, especially for T stage, reinforcing that restaging MRI is required to appropriately assess the frequent treatment-related tumor downstaging that follows NAT. Notably, the more advanced baseline radiological profile observed in the NAT cohort was an expected consequence of multidisciplinary treatment selection since patients with adverse baseline MRI features (e.g., more advanced T category, MRF involvement, tumor deposits) are preferentially triaged to NAT. Therefore, the non-NAT and NAT cohorts are not directly comparable exposure groups, and this is the reason why diagnostic performance was primarily described separately within each pathway.

A first relevant finding is that MRI exhibited good performance for dichotomized T staging, particularly in identifying tumors with advanced local extent. This is consistent with the current role of rectal MRI as the key imaging modality for local staging and restaging, as emphasized in recent ACR criteria and updated ESGAR recommendations [5,7]. However, our findings highlight that MRI-pathology concordance remains incomplete, even when categories are simplified into pragmatic clinically relevant groups.

In the overall cohort, T-stage agreement was 72.4%, with a sensitivity of 80.3% and a specificity of 67.0%. In the non-NAT subgroup, our findings were broadly in line with the Swedish population-based study by Dahlbäck et al., which included patients treated by primary surgery and reported sensitivity and specificity of 69% and 77% for differentiating T1-2 from T3-4, together with 42% and 81% for N1-2 detection [23]. They also fit with other studies, such as the OCUM trial, in which the MRI-based T category was correct in 63.5% of patients, overstaged in 22.9%, and understaged in 13.5% [24], and with a real-world surgical series of 114 patients without NAT reporting exact accuracies of 56.6% for T stage and 55.8% for N stage [25]. The better performance observed in our study may be explained by the use of dichotomized categories, which are more reproducible compared to granular TNM subclassification.

The observed predominance of T overstaging in our cohort is clinically plausible. MRI may overestimate extramural extension because desmoplastic reaction, fibrosis, edema, inflammatory changes, and acellular mucin can mimic residual or more extensive tumor [26,27]. This limitation is well recognized in both baseline staging and post-treatment assessment, although it is especially relevant after NAT [28]. By contrast, N staging remained more challenging. Although overall N-stage agreement was 73.0%, sensitivity was only 54.5% in the overall cohort and 44.4% in the non-NAT subgroup, whereas specificity remained higher. This is consistent with the longstanding difficulty of MRI-based nodal assessment in RC, which relies on indirect criteria (i.e., size, contour, internal signal, and treatment-related interval changes), none of which is sufficiently accurate in isolation [29]. Recent ESGAR recommendations have reinforced this concept by advocating a more structured and patient-level approach to cN and ycN assessment, rather than reliance on a single morphologic feature [6,7]. Our findings, thus, support that MRI is reasonably specific for nodal disease exclusion, but less reliable for sensitive detection of pathological involvement.

Our NAT restaging results are also similar to the available pooled evidence. A meta-analysis including 19 studies and 1262 patients reported a pooled sensitivity and specificity of 81% and 67% for MRI in restaging T3-T4 disease, and 77% for nodal restaging after chemoradiotherapy [30]. In our NAT restaging subgroup, sensitivity and specificity were 74.3% and 70.2% for T stage and 61.5% and 83.9% for N stage. Although our sensitivity for nodal disease was lower (and the specificity higher), the overall pattern remains comparable. Of note, the very poor agreement observed when baseline MRI was compared with final pathology in NAT patients should be interpreted as an expected consequence of treatment-related downstaging.

Exploratory analyses did not identify strong or reproducible predictors of MRI misclassification. Given the limited number of discordant cases, these regression analyses should be considered exploratory and hypothesis-generating, and the borderline associations observed should not be interpreted as stable independent predictors of MRI failure. For T-stage discordance, most baseline MRI variables were not consistently associated with error, and no stable multivariable model could be derived in the non-NAT subgroup. For N-stage misclassification, the most consistent signal was the association with anterior peritoneal reflection invasion, which is a known challenging structure in the assessment of RC [31]. However, the relationship of the latter with increased N-staging errors is not straightforward and might reflect greater interpretative uncertainty due to the anatomical complexity of certain tumors rather than a true independent predictor of MRI failure. Further studies with larger samples are needed to clarify this point.

Overall, our findings suggest that MRI discordance is unlikely to be explained by a single preoperative factor and is more plausibly related to the combined effects of tumor anatomy, treatment-induced tissue changes, and the intrinsic limitations of morphologic image interpretation in routine practice. From a practical perspective, these findings support the use of MRI as a risk-stratification tool rather than as a perfect surrogate for pathological TNM stage. Borderline T2/T3 findings, particularly when based on subtle extramural spiculation or suspected desmoplastic reaction, should be interpreted cautiously and reviewed in a multidisciplinary setting. Similarly, nodal downstaging after NAT should not be inferred from size reduction alone, because small residual nodes may still harbor tumor, whereas enlarged residual nodes may reflect fibrosis or mucinous degeneration without viable disease.

In the future, novel approaches such as MRI-based radiomics may help address some of these limitations, particularly in challenging areas such as nodal evaluation. In a recent systematic review and meta-analysis, we reported that MRI-based radiomics models showed overall moderate accuracy for predicting pathological nodal status in RC, although some individual studies reported substantially higher performance [32]. This potential is supported by other recent reviews and studies showing good external-validation performance for a multiparametric MRI deep-learning radiomics nomogram [33]. However, methodological heterogeneity, limited external validation, and the need for further standardization still preclude routine clinical implementation.

Our study has several strengths, namely a real-world design, pathological correlation of MRI findings, and separate analysis of non-NAT and NAT pathways. Its main limitations include the retrospective single-center design, modest sample size for modeling misclassification, and absence of formal multimodal response categories. In addition, the restriction to surgically treated patients is also relevant; since pathological staging of the surgical specimen was required as the reference standard, patients managed non-operatively (e.g., those entering watch-and-wait approaches) were not included. This may introduce selection bias and limits generalizability to the entire contemporary RC population. Another important limitation is the dichotomization of T and N categories; although this approach is common in previous MRI diagnostic accuracy studies and reflects clinically relevant decision points, it inevitably reduces staging granularity. Thus, our results should be interpreted as the performance of MRI for pragmatic binary risk stratification. Finally, some sources of variability, such as MRI field strength and formal interobserver variability, were not assessed. The absence of interobserver variability analysis is an important limitation because MRI staging is reader-dependent. However, the study was designed to evaluate the final MRI classification used in routine multidisciplinary care after double reading, rather than individual-reader accuracy.

## 5. Conclusions

This real-world study provides a contemporary estimate of the agreement between preoperative MRI and surgical pathology for dichotomized T and N staging in rectal cancer. Overall agreement was moderate, supporting MRI as a practical tool for multidisciplinary risk stratification, although not as a perfect surrogate for pathological locoregional staging. MRI performance was more consistent for identifying advanced T category than for detecting pathological nodal involvement. T-stage errors were predominantly related to overstaging and N-stage errors were more balanced between over- and understaging. Overall, these findings support the utility of MRI in contemporary rectal cancer management and highlight the importance of continued real-world monitoring, particularly for nodal assessment and persistent staging/restaging discrepancies. Future research should focus on improving nodal assessment, reducing persistent staging errors, and evaluating whether standardized reporting or quantitative imaging approaches can improve performance in routine practice.

## Figures and Tables

**Table 1:** Baseline characteristics of the study population. Data are presented as mean ± standard deviation or median [interquartile range], or n (%). *p* values compare the non-NAT and NAT cohorts. Continuous variables were compared using Student’s *t*-test or the Mann–Whitney U test, as appropriate according to distributional assumptions. Categorical variables were compared using the chi-square test or Fisher’s exact test.

Characteristic	Overall (n = 152)	Non-NAT (n = 70)	NAT (n = 82)	*p*-Value
Age, years	66.05 ± 10.19	68.06 ± 9.17	64.34 ± 10.74	0.023
Male sex	96 (63.2)	45 (64.3)	51 (62.2)	0.867
Tumor location/extent				0.001
Lower	4 (2.6)	2 (2.9)	2 (2.4)	
Lower + Mid	24 (15.8)	4 (5.7)	20 (24.4)	
Mid	35 (23.0)	18 (25.7)	17 (20.7)	
Mid + Upper	32 (21.1)	10 (14.3)	22 (26.8)	
Upper	57 (37.5)	36 (51.4)	21 (25.6)	
Mucinous component on baseline MRI	9 (5.9)	2 (2.9)	7 (8.5)	0.179
Tumor thickness on baseline MRI, mm	11.25 [8.00–15.00]	8.75 [6.12–12.38]	13.25 [10.00–16.75]	<0.001
MRF positive on baseline MRI	28 (18.4)	1 (1.4)	27 (32.9)	<0.001
EMVI on baseline MRI	29 (19.1)	5 (7.1)	24 (29.3)	<0.001
Tumor deposits on baseline MRI	6 (3.9)	0 (0.0)	6 (7.3)	0.031
Peritoneal reflection invasion on baseline MRI	15 (9.9)	4 (5.7)	11 (13.4)	0.172
Metastatic disease at baseline MRI	15 (9.9)	2 (2.9)	13 (15.9)	0.012

**Table 2:** Agreement and diagnostic performance of MRI for dichotomized T and N staging. Diagnostic performance metrics were calculated using surgical pathology as the reference standard. In the main analysis, MRI corresponded to staging MRI in the non-NAT cohort and restaging MRI in the NAT cohort. Overall agreement is presented as the proportion of correctly classified cases. Exact binomial 95% confidence intervals are provided for overall agreement, sensitivity, specificity, positive predictive value, and negative predictive value. Confidence intervals for Cohen’s kappa were calculated using standard asymptotic methods. McNemar’s test was used to assess directional disagreement between MRI-based and pathology-based classification. Agr, agreement; K, Cohen’s kappa; Sens, sensitivity; Sp, specificity; PPV, positive predictive value; NPV, negative predictive value.

Cohort	Agr (_95%_ CI)	K (_95%_ CI)	Sens (_95%_ CI)	Sp (_95%_ CI)	PPV (_95%_ CI)	NPV (_95%_ CI)	*p*-Val
Overall—T	72.4 (64.5–79.3)	0.452 (0.314–0.589)	80.3 (68.2–89.4)	67.0 (56.4–76.5)	62.0 (50.4–72.7)	83.6 (73.0–91.2)	0.009
Overall—N	73.0 (65.2–79.9)	0.349 (0.187–0.510)	54.5 (38.8–69.6)	80.6 (71.8–87.5)	53.3 (37.9–68.3)	81.3 (72.6–88.2)	1.000
non-NAT—T	72.9 (60.9–82.8)	0.473 (0.283–0.662)	88.5 (69.8–97.6)	63.6 (47.8–77.6)	59.0 (42.1–74.4)	90.3 (74.2–98.0)	0.006
non-NAT—N	68.6 (56.4–79.1)	0.206 (−0.040–0.453)	44.4 (21.5–69.2)	76.9 (63.2–87.5)	40.0 (19.1–63.9)	80.0 (66.3–90.0)	0.831
NAT—T	72.0 (60.9–81.3)	0.437 (0.244–0.631)	74.3 (56.7–87.5)	70.2 (55.1–82.7)	65.0 (48.3–79.4)	78.6 (63.2–89.7)	0.404
NAT—N	76.8 (66.2–85.4)	0.459 (0.252–0.667)	61.5 (40.6–79.8)	83.9 (71.7–92.4)	64.0 (42.5–82.0)	82.5 (70.1–91.3)	1.000

**Table 3:** Supplementary comparison of baseline MRI (bMRI) versus final pathology in the NAT cohort. Diagnostic performance metrics were calculated using surgical pathology as the reference standard. Overall agreement is presented as the proportion of correctly classified cases. Exact binomial 95% confidence intervals are provided for overall agreement, sensitivity, specificity, positive predictive value, and negative predictive value. Confidence intervals for Cohen’s kappa were calculated using standard asymptotic methods. McNemar’s test was used to assess directional disagreement between MRI-based and pathology-based classification.

Cohort	Agr (_95%_ CI)	K (_95%_ CI)	Sens (_95%_ CI)	Sp (_95%_ CI)	PPV (_95%_ CI)	NPV (_95%_ CI)	*p*-val
NAT bMRI—T	45.1 (34.1–56.5)	0.037 (−0.014–0.087)	100.0 (90.0–100.0)	4.3 (0.5–14.5)	43.8 (32.7–55.3)	100.0 (15.8–100.0)	<0.001
NAT bMRI—N	52.4 (41.1–63.6)	0.154 (−0.004–0.313)	80.8 (60.6–93.4)	39.3 (26.5–53.2)	38.2 (25.4–52.3)	81.5 (61.9–93.7)	<0.001

## Data Availability

Data are available upon reasonable request to the corresponding author. Sharing of imaging data may be subject to ethical and institutional restrictions and may require additional approval.

## Supplementary Materials

The following supporting information can be downloaded at: https://www.mdpi.com/article/10.3390/diagnostics16101481/s1, Table S1: Full baseline descriptive table; Table S2: Treatment-related variables in the NAT cohort; Table S3: Confusion matrices for dichotomized MRI versus pathology staging; Table S4: Bivariable analyses according to pathological T category; Table S5: Bivariable analyses according to pathological N category; Table S6: Bivariable analyses according to T misclassification; Table S7: Bivariable analyses according to N misclassification; Table S8: Univariable logistic regression for T misclassification; Table S9: Univariable logistic regression for N misclassification; Table S10: Cohort-specific multivariable models; Figure S1: Baseline vs. restaging MRI agreement in NAT; Figure S2: Forest-style plot of diagnostic performance for dichotomized T and N staging.
