# Supplementary material for: Diagnostic Performance and Misclassification Patterns of Preoperative MRI in Rectal Cancer: A Real-World Study
Source: Diagnostics (Basel). 2026 May 13;16(10):1481. doi: 10.3390/diagnostics16101481 (PMC13205548; doi:10.3390/diagnostics16101481)
Supplement: Supplementary file 1 [file diagnostics-16-01481-s001.zip › Supplementary Figure S1_legend.pdf]

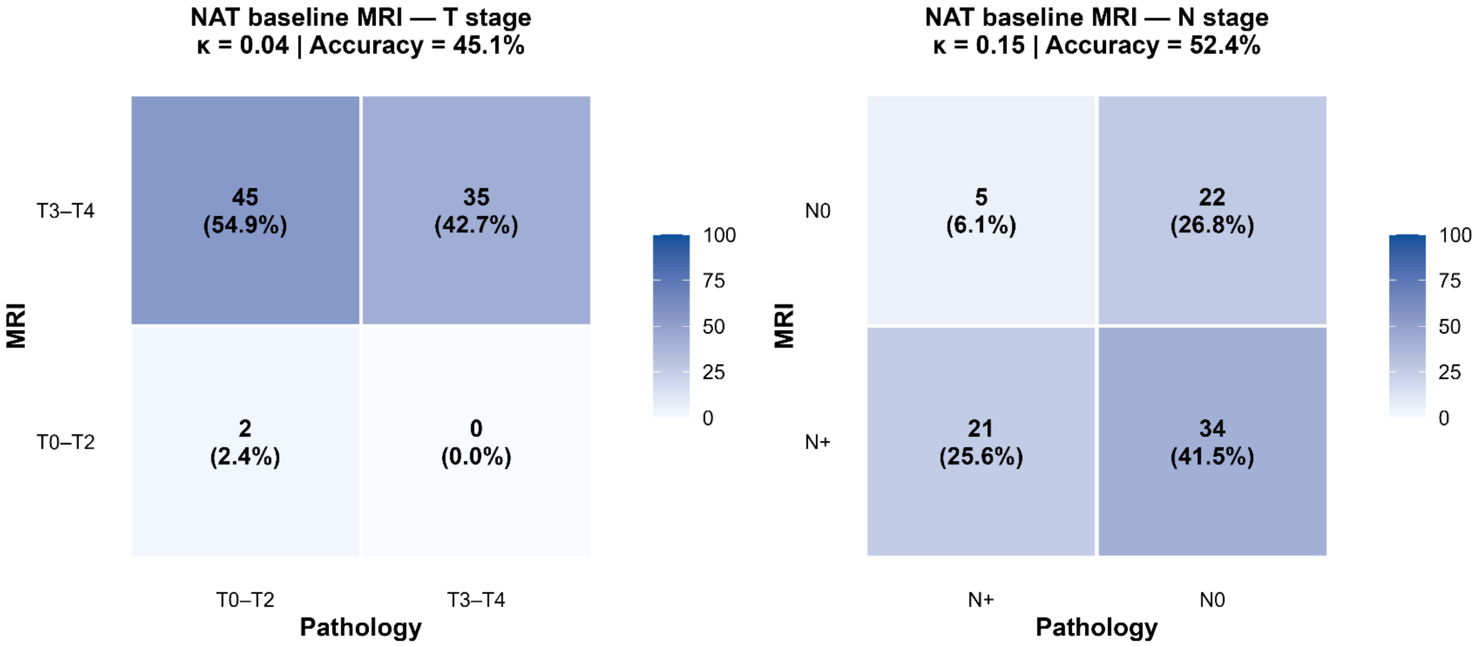

Supplementary Figure S1. Baseline vs restaging MRI agreement in NAT. Comparison of baseline MRI-based classification against final pathology for dichotomized T and N staging in NAT-treated patients.
