# Supplementary material for: Diagnostic Performance and Misclassification Patterns of Preoperative MRI in Rectal Cancer: A Real-World Study
Source: Diagnostics (Basel). 2026 May 13;16(10):1481. doi: 10.3390/diagnostics16101481 (PMC13205548; doi:10.3390/diagnostics16101481)
Supplement: Supplementary file 1 [file diagnostics-16-01481-s001.zip › Supplementary Figure S2_legend.pdf]

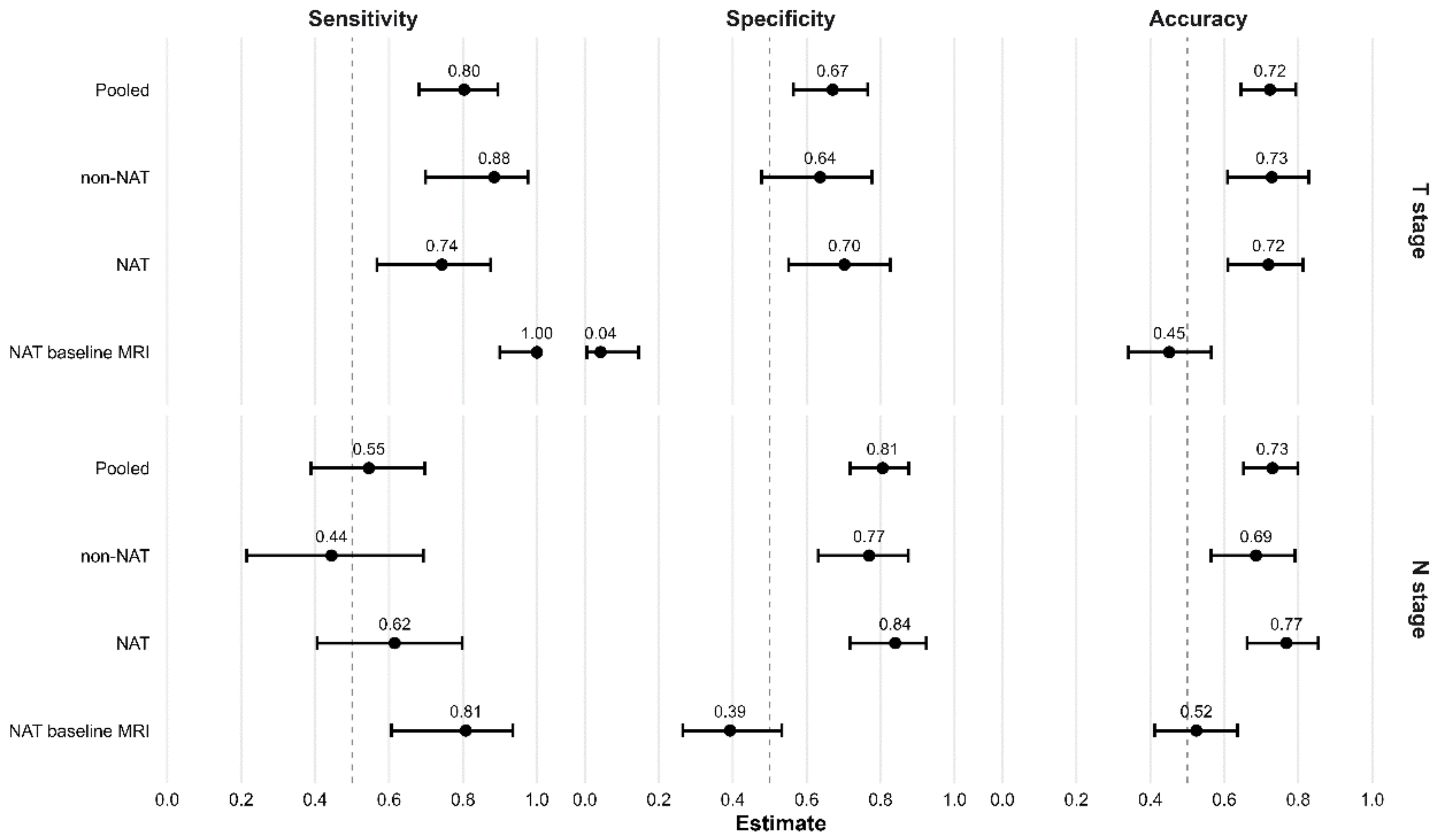

Supplementary Figure S2. Forest-style plot of diagnostic performance for dichotomized T and N staging. Dot-and-whisker plots show the diagnostic performance of MRI for dichotomized T and N staging, using surgical pathology as the reference standard. Results are presented separately for the pooled cohort, the non-NAT cohort, and the NAT cohort; supplementary results for baseline MRI in the NAT cohort are also shown. Metrics displayed include sensitivity, specificity, and accuracy. Horizontal lines represent 95% confidence intervals.
