# Supplementary material for: Diagnostic Performance and Misclassification Patterns of Preoperative MRI in Rectal Cancer: A Real-World Study
Source: Diagnostics (Basel). 2026 May 13;16(10):1481. doi: 10.3390/diagnostics16101481 (PMC13205548; doi:10.3390/diagnostics16101481)
Supplement: Supplementary file 1 [file diagnostics-16-01481-s001.zip › Supplementary Table S1.pdf]

**Characteristic**	**Overall (n = 152)**	**non-NAT (n = 70)**	**NAT (n = 82)**	**P value**
**Panel A. Demographic and tumor distribution**				
Age, years	66.05 ± 10.19	68.06 ± 9.17	64.34 ± 10.74	0.023
Male sex	96/152 (63.2)	45/70 (64.3)	51/82 (62.2)	0.867
Tumor location/extent				0.001
Lower	4/152 (2.6)	2/70 (2.9)	2/82 (2.4)	
Lower + mid	24/152 (15.8)	4/70 (5.7)	20/82 (24.4)	
Mid	35/152 (23.0)	18/70 (25.7)	17/82 (20.7)	
Mid + upper	32/152 (21.1)	10/70 (14.3)	22/82 (26.8)	
Upper	57/152 (37.5)	36/70 (51.4)	21/82 (25.6)	
**Panel B. Baseline MRI findings**				
Mucinous component on baseline MRI	9/152 (5.9)	2/70 (2.9)	7/82 (8.5)	0.179
Predominantly mucinous appearance on baseline MRI	13/152 (8.6)	2/70 (2.9)	11/82 (13.4)	0.022
Tumor thickness on baseline MRI, mm	11.25 [8.00–15.00]	8.75 [6.12–12.38]	13.25 [10.00–16.75]	<0.001
MRF positive on baseline MRI	28/152 (18.4)	1/70 (1.4)	27/82 (32.9)	<0.001
EMVI on baseline MRI	29/152 (19.1)	5/70 (7.1)	24/82 (29.3)	<0.001
EMVI extension on baseline MRI, mm	3.20 [2.50–4.30] (n = 29)	2.50 [2.00–2.90] (n = 5)	3.50 [2.95–4.43] (n = 24)	0.113
Tumor deposits on baseline MRI	6/152 (3.9)	0/70 (0.0)	6/82 (7.3)	0.031
Peritoneal reflection invasion on baseline MRI	15/152 (9.9)	4/70 (5.7)	11/82 (13.4)	0.172
Metastatic disease on baseline MRI*	15/152 (9.9)	2/70 (2.9)	13/82 (15.9)	0.012
Baseline MRI T stage				<0.001
T1-T2	33/152 (21.7)	31/70 (44.3)	2/82 (2.4)	
T3a	13/152 (8.6)	6/70 (8.6)	7/82 (8.5)	
T3b	56/152 (36.8)	24/70 (34.3)	32/82 (39.0)	
T3c	28/152 (18.4)	5/70 (7.1)	23/82 (28.0)	
T3d	4/152 (2.6)	0/70 (0.0)	4/82 (4.9)	
T4a	7/152 (4.6)	1/70 (1.4)	6/82 (7.3)	
T4b	11/152 (7.2)	3/70 (4.3)	8/82 (9.8)	
Baseline MRI N stage				<0.001

N0	77/152 (50.7)	50/70 (71.4)	27/82 (32.9)	
N1a	35/152 (23.0)	14/70 (20.0)	21/82 (25.6)	
N1b	13/152 (8.6)	4/70 (5.7)	9/82 (11.0)	
N1c	1/152 (0.7)	0/70 (0.0)	1/82 (1.2)	
N2a	21/152 (13.8)	2/70 (2.9)	19/82 (23.2)	
N2b	5/152 (3.3)	0/70 (0.0)	5/82 (6.1)	
**Panel C. Main MRI findings**				
Mucinous component on main MRI	12/152 (7.9)	2/70 (2.9)	10/82 (12.2)	0.038
Predominantly mucinous appearance on main MRI	22/152 (14.5)	2/70 (2.9)	20/82 (24.4)	<0.001
MRF positive on main MRI	14/152 (9.2)	1/70 (1.4)	13/82 (15.9)	0.002
EMVI on main MRI	16/152 (10.5)	5/70 (7.1)	11/82 (13.4)	0.290
EMVI extension on main MRI, mm	3.40 [2.50–4.12] (n = 16)	2.50 [2.00–2.90] (n = 5)	3.70 [2.95–4.50] (n = 11)	0.103
Tumor deposits on main MRI	3/152 (2.0)	0/70 (0.0)	3/82 (3.7)	0.250
Peritoneal reflection invasion on main MRI	8/152 (5.3)	4/70 (5.7)	4/82 (4.9)	1.000
Main MRI T stage				0.054
T0	5/152 (3.3)	0/70 (0.0)	5/82 (6.1)	
T1-T2	68/152 (44.7)	31/70 (44.3)	37/82 (45.1)	
T3a	16/152 (10.5)	6/70 (8.6)	10/82 (12.2)	
T3b	36/152 (23.7)	24/70 (34.3)	12/82 (14.6)	
T3c	13/152 (8.6)	5/70 (7.1)	8/82 (9.8)	
T3d	2/152 (1.3)	0/70 (0.0)	2/82 (2.4)	
T4a	4/152 (2.6)	1/70 (1.4)	3/82 (3.7)	
T4b	8/152 (5.3)	3/70 (4.3)	5/82 (6.1)	
Main MRI N stage				0.650
N0	107/152 (70.4)	50/70 (71.4)	57/82 (69.5)	
N1a	30/152 (19.7)	14/70 (20.0)	16/82 (19.5)	
N1b	7/152 (4.6)	4/70 (5.7)	3/82 (3.7)	
N1c	2/152 (1.3)	0/70 (0.0)	2/82 (2.4)	
N2a	6/152 (3.9)	2/70 (2.9)	4/82 (4.9)	
Main MRI to pathology interval, days	35.0 [26.0–49.3]	44.5 [33.0–54.0]	28.0 [20.0–41.8]	<0.001
**Panel D. Pathology findings**				

Mucinous component on pathology	12/152 (7.9)	2/70 (2.9)	10/82 (12.2)	0.038
Predominantly mucinous component on pathology	35/152 (23.0)	12/70 (17.1)	23/82 (28.0)	0.126
Tumor deposits on pathology	16/152 (10.5)	7/70 (10.0)	9/82 (11.0)	1.000
CRM positive on pathology	3/152 (2.0)	0/70 (0.0)	3/82 (3.7)	0.250
Distance to CRM on pathology, mm	17.00 [7.93–21.00] (n = 78)	17.50 [8.00–21.00] (n = 34)	15.50 [6.75–20.25] (n = 44)	0.535
Lymphovascular invasion on pathology	23/152 (15.1)	8/70 (11.4)	15/82 (18.3)	0.265
Perineural invasion on pathology	24/152 (15.8)	6/70 (8.6)	18/82 (22.0)	0.027
Tumor budding on pathology				0.979
Grade 0	131/152 (86.2)	60/70 (85.7)	71/82 (86.6)	
Grade 1	15/152 (9.9)	7/70 (10.0)	8/82 (9.8)	
Grade 2	6/152 (3.9)	3/70 (4.3)	3/82 (3.7)	
Pathological T stage				0.455
T0	17/152 (11.2)	6/70 (8.6)	11/82 (13.4)	
T1	19/152 (12.5)	11/70 (15.7)	8/82 (9.8)	
T2	55/152 (36.2)	27/70 (38.6)	28/82 (34.1)	
T3	56/152 (36.8)	25/70 (35.7)	31/82 (37.8)	
T4a	3/152 (2.0)	0/70 (0.0)	3/82 (3.7)	
T4b	2/152 (1.3)	1/70 (1.4)	1/82 (1.2)	
Pathological N stage				0.326
N0	108/152 (71.1)	52/70 (74.3)	56/82 (68.3)	
N1a	11/152 (7.2)	5/70 (7.1)	6/82 (7.3)	
N1b	9/152 (5.9)	2/70 (2.9)	7/82 (8.5)	
N1c	12/152 (7.9)	5/70 (7.1)	7/82 (8.5)	
N2a	8/152 (5.3)	5/70 (7.1)	3/82 (3.7)	
N2b	3/152 (2.0)	0/70 (0.0)	3/82 (3.7)	
N2c	1/152 (0.7)	1/70 (1.4)	0/82 (0.0)	

Supplementary Table S1. Full baseline descriptive table. Data are presented as mean ± standard deviation, median [interquartile range], or n/N (%), as appropriate. Percentages were calculated using available data for each variable; therefore, denominators may vary across rows. P values compare the non-NAT and NAT cohorts and were calculated using Student's t-test for age, Mann–Whitney U test for non-normally distributed continuous variables, Fisher's exact test for binary variables, and chi-square test for multi-category variables. *Location of metastases in overall

cohort: liver (8), lung (6), external iliac nodes (2). CRM, circunferencial resection margin. MRF, mesorectal fascia; EMVI, extramural vascular invasion.
