# Supplementary material for: Diagnostic Performance and Misclassification Patterns of Preoperative MRI in Rectal Cancer: A Real-World Study
Source: Diagnostics (Basel). 2026 May 13;16(10):1481. doi: 10.3390/diagnostics16101481 (PMC13205548; doi:10.3390/diagnostics16101481)
Supplement: Supplementary file 1 [file diagnostics-16-01481-s001.zip › Supplementary Table S2.pdf]

**Characteristic**	**NAT (n = 82)**
Radiotherapy during treatment	22/82 (26.8)
**Neoadjuvant chemotherapy**	
No chemotherapy	39/82 (47.6)
Capecitabine	14/82 (17.1)
FOLFOX, XELOX or other	29/82 (35.4)
Interval metastatic surgery during treatment	11/82 (13.4)
Baseline MRI to treatment interval, days	66.0 [42.5–142.5]
Treatment to restaging MRI interval, days	49.0 [40.0–56.0]
Baseline MRI to restaging MRI interval, days	113.0 [91.0–184.8]
Main MRI to pathology interval, days	28.0 [20.0–41.8]

Supplementary Table S2. Treatment-related variables in the NAT cohort. Categorical variables are presented as n/N (%), and continuous variables as median [interquartile range].
