# Supplementary material for: Diagnostic Performance and Misclassification Patterns of Preoperative MRI in Rectal Cancer: A Real-World Study
Source: Diagnostics (Basel). 2026 May 13;16(10):1481. doi: 10.3390/diagnostics16101481 (PMC13205548; doi:10.3390/diagnostics16101481)
Supplement: Supplementary file 1 [file diagnostics-16-01481-s001.zip › Supplementary Table S3.pdf]

	Overall cohort			Non-NAT cohort			NAT cohort (Restaging)			NAT cohort (baseline)		
**MRI / Pathology**	**T0–T2**	**T3–T4**	**Total**	**T0–T2**	**T3–T4**	**Total**	**T0–T2**	**T3–T4**	**Total**	**T0–T2**	**T3–T4**	**Total**
**T0–T2**	61	12	73	28	3	31	33	9	42	2	0	2
**T3–T4**	30	49	79	16	23	39	14	26	40	45	35	80
**Total**	91	61	152	44	26	70	47	35	82	47	35	82
**MRI / Pathology**	**N0**	**N+**	**Total**	**N0**	**N+**	**Total**	**N0**	**N+**	**Total**	**N0**	**N+**	**Total**
**N0**	87	20	107	40	10	50	47	10	57	22	5	27
**N+**	21	24	45	12	8	20	9	16	25	34	21	55
**Total**	108	44	152	52	18	70	56	26	82	56	26	82

Supplementary Table S3. Confusion matrices for dichotomized MRI versus pathology staging. Rows correspond to MRI classification and columns correspond to pathology. In the main analyses, MRI corresponded to staging MRI in the non-NAT cohort and restaging MRI in the NAT cohort. The last column group for the NAT cohort refers to the supplementary baseline MRI analysis.
