# Supplementary material for: Diagnostic Performance and Misclassification Patterns of Preoperative MRI in Rectal Cancer: A Real-World Study
Source: Diagnostics (Basel). 2026 May 13;16(10):1481. doi: 10.3390/diagnostics16101481 (PMC13205548; doi:10.3390/diagnostics16101481)
Supplement: Supplementary file 1 [file diagnostics-16-01481-s001.zip › Supplementary Table S4.pdf]

**Characteristic**	**Pathological T0–T2 (n = 91)**	**Pathological T3–T4 (n = 61)**	**P value**
Sex			0.613
Female	35 (38.5)	21 (34.4)	
Male	56 (61.5)	40 (65.6)	
Age, years	67.00 [59.00–74.50]	66.00 [59.00–72.00]	0.544
Cohort			0.487
NAT	47 (51.6)	35 (57.4)	
non-NAT	44 (48.4)	26 (42.6)	
Tumor location/extent			0.006
Lower	2 (2.2)	2 (3.3)	
Lower + Mid	16 (17.6)	8 (13.1)	
Mid	29 (31.9)	6 (9.8)	
Mid + Upper	18 (19.8)	14 (23.0)	
Upper	26 (28.6)	31 (50.8)	
Mucinous component on baseline MRI			0.030
No	89 (97.8)	54 (88.5)	
Yes	2 (2.2)	7 (11.5)	
Mucinous composition on baseline MRI			0.025
No	87 (95.6)	52 (85.2)	
Yes	4 (4.4)	9 (14.8)	
Tumor thickness on baseline MRI, mm	11.50 [7.15–15.00]	11.00 [9.50–16.00]	0.145
MRF positive on baseline MRI			0.042
No	79 (86.8)	45 (73.8)	
Yes	12 (13.2)	16 (26.2)	
EMVI on baseline MRI			<0.001
No	82 (90.1)	41 (67.2)	
Yes	9 (9.9)	20 (32.8)	
EMVI extension on baseline MRI, mm*	2.80 [2.00–3.50]	3.75 [2.98–4.97]	0.028
Tumor deposits on baseline MRI			0.039
No	90 (98.9)	56 (91.8)	
Yes	1 (1.1)	5 (8.2)	
Peritoneal reflection invasion on baseline MRI			<0.001

No	88 (96.7)	49 (80.3)	
Yes	3 (3.3)	12 (19.7)	
Metastatic disease on baseline MRI			0.027
No	86 (94.5)	51 (83.6)	
Yes	5 (5.5)	10 (16.4)	

Supplementary Table S4. Bivariable analyses according to pathological T category. Data are presented as median [interquartile range] or n (%), as appropriate. P values were obtained using Student's t-test or the Mann–Whitney U test for continuous variables, as appropriate, and using the chi-square test or Fisher's exact test for categorical variables, as appropriate. *EMVI extension was assessed only in patients with measurable EMVI extension on baseline MRI. EMVI, extramural vascular invasion. MRF, mesorectal fascia.
