# Supplementary material for: Diagnostic Performance and Misclassification Patterns of Preoperative MRI in Rectal Cancer: A Real-World Study
Source: Diagnostics (Basel). 2026 May 13;16(10):1481. doi: 10.3390/diagnostics16101481 (PMC13205548; doi:10.3390/diagnostics16101481)
Supplement: Supplementary file 1 [file diagnostics-16-01481-s001.zip › Supplementary Table S5.pdf]

**Characteristic**	**Pathological N0 (n = 108)**	**Pathological N+ (n = 44)**	**P value**
Sex			0.412
Female	42 (38.9)	14 (31.8)	
Male	66 (61.1)	30 (68.2)	
Age, years	66.00 [57.00–72.25]	67.50 [60.00–73.25]	0.262
Cohort			0.417
NAT	56 (51.9)	26 (59.1)	
non-NAT	52 (48.1)	18 (40.9)	
Tumor location/extent			0.014
Lower	2 (1.9)	2 (4.5)	
Lower + Mid	16 (14.8)	8 (18.2)	
Mid	32 (29.6)	3 (6.8)	
Mid + Upper	23 (21.3)	9 (20.5)	
Upper	35 (32.4)	22 (50.0)	
Mucinous component on baseline MRI			0.719
No	102 (94.4)	41 (93.2)	
Yes	6 (5.6)	3 (6.8)	
Predominantly mucinous appearance on baseline MRI			0.523
No	100 (92.6)	39 (88.6)	
Yes	8 (7.4)	5 (11.4)	
Tumor thickness on baseline MRI, mm	11.50 [8.00–15.00]	10.75 [9.00–16.00]	0.613
MRF positive on baseline MRI			0.024
No	93 (86.1)	31 (70.5)	
Yes	15 (13.9)	13 (29.5)	
EMVI on baseline MRI			<0.001
No	97 (89.8)	26 (59.1)	
Yes	11 (10.2)	18 (40.9)	
EMVI extension on baseline MRI, mm*	2.90 [2.25–3.75]	3.75 [3.00–4.72]	0.183
Tumor deposits on baseline MRI			0.357
No	105 (97.2)	41 (93.2)	

Yes	3 (2.8)	3 (6.8)	
Peritoneal reflection invasion on baseline MRI			0.136
No	100 (92.6)	37 (84.1)	
Yes	8 (7.4)	7 (15.9)	
Metastatic disease on baseline MRI			0.013
No	102 (94.4)	35 (79.5)	
Yes	6 (5.6)	9 (20.5)	

Supplementary Table S5. Bivariable analyses according to pathological N category. Data are presented as median [interquartile range] or n (%), as appropriate. P values were obtained using Student's t-test or the Mann–Whitney U test for continuous variables, as appropriate, and using the chi-square test for categorical variables, or Fisher's exact test when appropriate. *EMVI extension was assessed only in patients with measurable EMVI extension on baseline MRI.
