# Supplementary material for: Diagnostic Performance and Misclassification Patterns of Preoperative MRI in Rectal Cancer: A Real-World Study
Source: Diagnostics (Basel). 2026 May 13;16(10):1481. doi: 10.3390/diagnostics16101481 (PMC13205548; doi:10.3390/diagnostics16101481)
Supplement: Supplementary file 1 [file diagnostics-16-01481-s001.zip › Supplementary Table S6.pdf]

Overall cohort			
**Characteristic**	**No T misclassification (n = 110)**	**T misclassification (n = 42)**	**P value**
Sex			0.859
Female	41 (37.3)	15 (35.7)	
Male	69 (62.7)	27 (64.3)	
Age, years	67.00 [61.00–73.00]	64.50 [55.25–71.75]	0.188
Cohort			0.901
NAT	59 (53.6)	23 (54.8)	
non-NAT	51 (46.4)	19 (45.2)	
Tumor location/extent			0.551
Lower	2 (1.8)	2 (4.8)	
Lower + Mid	18 (16.4)	6 (14.3)	
Mid	28 (25.5)	7 (16.7)	
Mid + Upper	21 (19.1)	11 (26.2)	
Upper	41 (37.3)	16 (38.1)	
Mucinous component on baseline MRI			0.708
No	104 (94.5)	39 (92.9)	
Yes	6 (5.5)	3 (7.1)	
Predominantly mucinous appearance on baseline MRI			1.000
No	100 (90.9)	39 (92.9)	
Yes	10 (9.1)	3 (7.1)	
Tumor thickness on baseline MRI, mm	11.00 [8.50–15.75]	12.50 [8.00–15.00]	0.867
MRF positive on baseline MRI			0.554
No	91 (82.7)	33 (78.6)	
Yes	19 (17.3)	9 (21.4)	
EMVI on baseline MRI			0.995
No	89 (80.9)	34 (81.0)	
Yes	21 (19.1)	8 (19.0)	
EMVI extension on baseline MRI, mm*	3.50 [2.50–4.50]	3.15 [2.88–3.62]	0.695
Tumor deposits on baseline MRI			0.348

No	107 (97.3)	39 (92.9)	
Yes	3 (2.7)	3 (7.1)	
Peritoneal reflection invasion on baseline MRI			1.000
No	99 (90.0)	38 (90.5)	
Yes	11 (10.0)	4 (9.5)	
Metastatic disease on baseline MRI			0.360
No	101 (91.8)	36 (85.7)	
Yes	9 (8.2)	6 (14.3)	
Main MRI to pathology interval, days	33.5 [23.0–49.0]	38.5 [28.5–59.3]	0.107
**non-NAT cohort**			
**Characteristic**	**No T misclassification (n = 51)**	**T misclassification (n = 19)**	**P value**
Sex			0.904
Female	18 (35.3)	7 (36.8)	
Male	33 (64.7)	12 (63.2)	
Age, years	68.00 [62.00–73.00]	68.00 [63.00–77.50]	0.830
Tumor location/extent			0.587
Lower	1 (2.0)	1 (5.3)	
Lower + Mid	2 (3.9)	2 (10.5)	
Mid	13 (25.5)	5 (26.3)	
Mid + Upper	7 (13.7)	3 (15.8)	
Upper	28 (54.9)	8 (42.1)	
Mucinous component on baseline MRI			0.472
No	50 (98.0)	18 (94.7)	
Yes	1 (2.0)	1 (5.3)	
Predominantly mucinous appearance on baseline MRI			0.472
No	50 (98.0)	18 (94.7)	
Yes	1 (2.0)	1 (5.3)	
Tumor extension on baseline MRI, cm	10.00 [7.00–12.00]	7.50 [5.50–12.75]	0.331
MRF positive on baseline MRI			1.000
No	50 (98.0)	19 (100.0)	
Yes	1 (2.0)	0 (0.0)	
EMVI on baseline MRI			0.313

No	46 (90.2)	19 (100.0)	
Yes	5 (9.8)	0 (0.0)	
EMVI extension on baseline MRI, mm*	2.50 [2.00–2.90]	—	—
Tumor deposits on baseline MRI			—
No	51 (100.0)	19 (100.0)	
Peritoneal reflection invasion on baseline MRI			0.568
No	47 (92.2)	19 (100.0)	
Yes	4 (7.8)	0 (0.0)	
Metastatic disease on baseline MRI			1.000
No	49 (96.1)	18 (100.0)	
Yes	2 (3.9)	0 (0.0)	
Main MRI to pathology interval, days	42.0 [29.5–51.5]	48.0 [42.0–62.0]	0.056
**NAT cohort**			
**Characteristic**	**No T misclassification (n = 59)**	**T misclassification (n = 23)**	**P value**
Sex			0.725
Female	23 (39.0)	8 (34.8)	
Male	36 (61.0)	15 (65.2)	
Age, years	67.00 [58.50–73.50]	60.00 [54.50–66.50]	0.043
Tumor location/extent			0.224
Lower	1 (1.7)	1 (4.3)	
Lower + Mid	16 (27.1)	4 (17.4)	
Mid	15 (25.4)	2 (8.7)	
Mid + Upper	14 (23.7)	8 (34.8)	
Upper	13 (22.0)	8 (34.8)	
Mucinous component on baseline MRI			1.000
No	54 (91.5)	21 (91.3)	
Yes	5 (8.5)	2 (8.7)	
Predominantly mucinous appearance on baseline MRI			0.720
No	50 (84.7)	21 (91.3)	
Yes	9 (15.3)	2 (8.7)	
Tumor extension on baseline MRI, cm	13.00 [9.75–16.50]	14.00 [12.15–16.50]	0.313
MRF positive on baseline MRI			0.455
No	41 (69.5)	14 (60.9)	

Yes	18 (30.5)	9 (39.1)	
EMVI on baseline MRI			0.493
No	43 (72.9)	15 (65.2)	
Yes	16 (27.1)	8 (34.8)	
EMVI extension on baseline MRI, mm*	4.00 [2.95–4.97]	3.15 [2.88–3.62]	0.200
Tumor deposits on baseline MRI			0.342
No	56 (94.9)	20 (87.0)	
Yes	3 (5.1)	3 (13.0)	
Peritoneal reflection invasion on baseline MRI			0.493
No	52 (88.1)	19 (82.6)	
Yes	7 (11.9)	4 (17.4)	
Metastatic disease on baseline MRI			0.175
No	52 (88.1)	17 (73.9)	
Yes	7 (11.9)	6 (26.1)	
Main MRI to pathology interval, days	27.0 [20.0–41.5]	30.0 [24.0–39.5]	0.290

Supplementary Table S6. Bivariable analyses according to T misclassification. Data are presented as median [interquartile range] or n (%), as appropriate. P values were obtained using Student's t-test or Mann–Whitney U test for continuous variables, and chi-square or Fisher's exact test for categorical variables, as appropriate. *EMVI extension on baseline MRI was assessed only in patients with measurable EMVI extension; in the non-NAT cohort, no summary estimate could be derived for the misclassified group because no patient in that group had measurable EMVI extension.
