# Supplementary material for: Diagnostic Performance and Misclassification Patterns of Preoperative MRI in Rectal Cancer: A Real-World Study
Source: Diagnostics (Basel). 2026 May 13;16(10):1481. doi: 10.3390/diagnostics16101481 (PMC13205548; doi:10.3390/diagnostics16101481)
Supplement: Supplementary file 1 [file diagnostics-16-01481-s001.zip › Supplementary Table S7.pdf]

Overall cohort			
**Characteristic**	**No N misclassification (n = 111)**	**N misclassification (n = 41)**	**P value**
Sex			0.968
Female	41 (36.9)	15 (36.6)	
Male	70 (63.1)	26 (63.4)	
Age, years	66.00 [57.00–72.50]	67.00 [62.00–73.00]	0.207
Cohort			0.253
NAT	63 (56.8)	19 (46.3)	
non-NAT	48 (43.2)	22 (53.7)	
Tumor location/extent			0.072
Lower	3 (2.7)	1 (2.4)	
Lower + Mid	20 (18.0)	4 (9.8)	
Mid	29 (26.1)	6 (14.6)	
Mid + Upper	25 (22.5)	7 (17.1)	
Upper	34 (30.6)	23 (56.1)	
Mucinous component on baseline MRI			1.000
No	104 (93.7)	39 (95.1)	
Yes	7 (6.3)	2 (4.9)	
Predominantly mucinous appearance on baseline MRI			0.515
No	100 (90.1)	39 (95.1)	
Yes	11 (9.9)	2 (4.9)	
Tumor thickness on baseline MRI, mm	11.50 [8.00–15.00]	11.00 [9.00–16.00]	0.676
MRF positive on baseline MRI			0.794
No	90 (81.1)	34 (82.9)	
Yes	21 (18.9)	7 (17.1)	
EMVI on baseline MRI			0.584
No	91 (82.0)	32 (78.0)	
Yes	20 (18.0)	9 (22.0)	
EMVI extension on baseline MRI, mm*	3.60 [2.50–4.75]	3.10 [2.90–3.50]	0.421
Tumor deposits on baseline MRI			1.000
No	106 (95.5)	40 (97.6)	
Yes	5 (4.5)	1 (2.4)	

Peritoneal reflection invasion on baseline MRI			0.028
No	104 (93.7)	33 (80.5)	
Yes	7 (6.3)	8 (19.5)	
Metastatic disease on baseline MRI			1.000
No	100 (90.1)	37 (90.2)	
Yes	11 (9.9)	4 (9.8)	
Main MRI to pathology interval, days	34.0 [23.0–49.0]	40.0 [28.0–51.0]	0.414
**Non-NAT cohort**			
**Characteristic**	**No N misclassification (n = 48)**	**N misclassification (n = 22)**	**P value**
Sex			0.645
Female	18 (37.5)	7 (31.8)	
Male	30 (62.5)	15 (68.2)	
Age, years	68.00 [63.75–76.25]	67.50 [60.50–72.50]	0.600
Tumor location/extent			0.590
Lower	1 (2.1)	1 (4.5)	
Lower + Mid	3 (6.2)	1 (4.5)	
Mid	14 (29.2)	4 (18.2)	
Mid + Upper	8 (16.7)	2 (9.1)	
Upper	22 (45.8)	14 (63.6)	
Mucinous component on baseline MRI			1.000
No	46 (95.8)	22 (100.0)	
Yes	2 (4.2)	0 (0.0)	
Predominantly mucinous appearance on baseline MRI			1.000
No	46 (95.8)	22 (100.0)	
Yes	2 (4.2)	0 (0.0)	
Tumor thickness on baseline MRI, mm	8.25 [5.95–12.00]	10.50 [7.35–13.38]	0.126
MRF positive on baseline MRI			0.314
No	48 (100.0)	21 (95.5)	
Yes	0 (0.0)	1 (4.5)	
EMVI on baseline MRI			0.646
No	45 (93.8)	20 (90.9)	
Yes	3 (6.2)	2 (9.1)	

EMVI extension on baseline MRI, mm*	2.50 [2.25–3.50]	2.45 [2.23–2.67]	1.000
Tumor deposits on baseline MRI			—
No	48 (100.0)	22 (100.0)	
Peritoneal reflection invasion on baseline MRI			0.089
No	47 (97.9)	19 (86.4)	
Yes	1 (2.1)	3 (13.6)	
Metastatic disease on baseline MRI			1.000
No	46 (95.8)	22 (100.0)	
Yes	2 (4.2)	0 (0.0)	
Main MRI to pathology interval, days	43.5 [33.0–53.8]	46.0 [33.0–54.0]	0.885
**NAT cohort**			
**Characteristic**	**No N misclassification (n = 63)**	**N misclassification (n = 19)**	**P value**
Sex			0.659
Female	23 (36.5)	8 (42.1)	
Male	40 (63.5)	11 (57.9)	
Age, years	64.00 [55.50–70.00]	67.00 [63.00–73.00]	0.061
Tumor location/extent			0.172
Lower	2 (3.2)	0 (0.0)	
Lower + Mid	17 (27.0)	3 (15.8)	
Mid	15 (23.8)	2 (10.5)	
Mid + Upper	17 (27.0)	5 (26.3)	
Upper	12 (19.0)	9 (47.4)	
Mucinous component on baseline MRI			0.660
No	58 (92.1)	17 (89.5)	
Yes	5 (7.9)	2 (10.5)	
Predominantly mucinous appearance on baseline MRI			1.000
No	54 (85.7)	17 (89.5)	
Yes	9 (14.3)	2 (10.5)	
Tumor thickness on baseline MRI, mm	13.50 [10.00–16.50]	13.00 [9.75–17.50]	0.783
MRF positive on baseline MRI			0.887
No	42 (66.7)	13 (68.4)	
Yes	21 (33.3)	6 (31.6)	

EMVI on baseline MRI			0.408
No	46 (73.0)	12 (63.2)	
Yes	17 (27.0)	7 (36.8)	
EMVI extension on baseline MRI, mm*	4.00 [2.80–5.50]	3.50 [3.05–3.75]	0.260
Tumor deposits on baseline MRI			1.000
No	58 (92.1)	18 (94.7)	
Yes	5 (7.9)	1 (5.3)	
Peritoneal reflection invasion on baseline MRI			0.116
No	57 (90.5)	14 (73.7)	
Yes	6 (9.5)	5 (26.3)	
Metastatic disease on baseline MRI			0.486
No	54 (85.7)	15 (78.9)	
Yes	9 (14.3)	4 (21.1)	
Main MRI to pathology interval, days	28.0 [20.0–40.5]	28.0 [20.0–45.5]	0.586

Supplementary Table S7. Bivariable analyses according to N misclassification. Data are presented as median [interquartile range] or n (%), as appropriate. P values were obtained using Student’s t-test or Mann–Whitney U test for continuous variables, and chi-square or Fisher’s exact test for categorical variables, as appropriate. *EMVI extension on baseline MRI was assessed only in patients with measurable EMVI extension. NAT-specific treatment and interval variables are reported separately in Supplementary Table S10.
