# Supplementary material for: Diagnostic Performance and Misclassification Patterns of Preoperative MRI in Rectal Cancer: A Real-World Study
Source: Diagnostics (Basel). 2026 May 13;16(10):1481. doi: 10.3390/diagnostics16101481 (PMC13205548; doi:10.3390/diagnostics16101481)
Supplement: Supplementary file 1 [file diagnostics-16-01481-s001.zip › Supplementary Table S8.pdf]

Overall cohort					
**Predictor**	**n**	**Events**	**OR**	**95% CI**	**P value**
Male sex	152	42	1.07	0.51–2.28	0.859
Age, per year	152	42	0.98	0.94–1.01	0.188
NAT cohort (vs non-NAT)	152	42	1.05	0.51–2.15	0.901
Tumor location/extent: lower (vs upper)	152	42	2.56	0.29–22.88	0.367
Tumor location/extent: lower + mid (vs upper)	152	42	0.85	0.27-2.46	0.777
Tumor location/extent: mid (vs upper)	152	42	0.64	0.22-1.71	0.387
Tumor location/extent: mid + upper (vs upper)	152	42	1.34	0.52-3.40	0.535
Mucinous component on baseline MRI	152	42	1.33	0.27–5.32	0.694
Predominantly mucinous appearance on baseline MRI	152	42	0.77	0.17–2.67	0.702
Tumor thickness on baseline MRI, per mm	152	42	0.99	0.94–1.03	0.665
MRF positive on baseline MRI	152	42	1.31	0.52–3.11	0.555
EMVI on baseline MRI	152	42	1.00	0.38–2.40	0.995
EMVI extension on baseline MRI, per mm	29	8	0.78	0.39–1.35	0.416
Tumor deposits on baseline MRI	152	42	2.74	0.49–15.37	0.228
Peritoneal reflection invasion on baseline MRI	152	42	0.95	0.25–2.96	0.930
Metastatic disease on baseline MRI	152	42	1.87	0.59–5.56	0.265
Main MRI to pathology interval, per day	152	42	1.01	0.99-1.02	0.295
**non-NAT cohort**					
**Predictor**	**n**	**Events**	**OR**	**95% CI**	**P value**
Male sex	70	19	0.94	0.32–2.90	0.904
Age, per year	70	19	1.01	0.95–1.07	0.815
Tumor location/extent: lower (vs upper)	70	19	3.50	0.13-95.47	0.394
Tumor location/extent: lower + mid (vs upper)	70	19	3.50	0.37-33.23	0.245
Tumor location/extent: mid (vs upper)	70	19	1.35	0.35-4.88	0.653
Tumor location/extent: mid+upper (vs upper)	70	19	1.50	0.28-6.90	0.611

Mucinous component on baseline MRI	70	19	2.78	0.11–72.75	0.478
Predominantly mucinous appearance on baseline MRI	70	19	2.78	0.11–72.75	0.478
Tumor thickness on baseline MRI, per mm	70	19	0.97	0.87–1.07	0.572
MRF positive on baseline MRI	70	19	NE	NE	NE
EMVI on baseline MRI	70	19	NE	NE	NE
EMVI extension on baseline MRI, per mm	5	0	NE	NE	NE
Tumor deposits on baseline MRI	70	19	NE	NE	NE
Peritoneal reflection invasion on baseline MRI	70	19	NE	NE	NE
Metastatic disease on baseline MRI	70	19	NE	NE	NE
Main MRI to pathology interval, per day	70	19	1.03	1.00-1.07	0.057
**NAT cohort**					
**Predictor**	**n**	**Events**	**OR**	**95% CI**	**P value**
Male sex	82	23	1.20	0.45–3.39	0.725
Age, per year	82	23	0.96	0.91–1.00	0.069
Tumor location/extent: lower (vs upper)	82	23	1.63	0.06–45.14	0.744
Tumor location/extent: lower + mid (vs upper)	82	23	0.41	0.09–1.60	0.209
Tumor location/extent: mid (vs upper)	82	23	0.22	0.03–1.05	0.081
Tumor location/extent: mid + upper (vs upper)	82	23	0.93	0.27–3.23	0.907
Mucinous component on baseline MRI	82	23	1.03	0.14–5.19	0.974
Predominantly mucinous appearance on baseline MRI	82	23	0.53	0.08–2.28	0.440
Tumor thickness on baseline MRI, per mm	82	23	0.99	0.93–1.04	0.777
MRF positive on baseline MRI	82	23	1.46	0.53–3.98	0.457
EMVI on baseline MRI	82	23	1.43	0.50–3.99	0.494
EMVI extension on baseline MRI, per mm	24	8	0.67	0.31–1.21	0.242
Tumor deposits on baseline MRI	82	23	2.80	0.48–16.23	0.230
Peritoneal reflection invasion on baseline MRI	82	23	1.56	0.38–5.80	0.512
Metastatic disease on baseline MRI	82	23	2.62	0.75–8.99	0.122

Baseline MRI to treatment interval, per day	82	23	1.00	1.00–1.01	0.529
Treatment to restaging MRI interval, per day	82	23	1.00	0.97–1.03	0.929
Baseline MRI to restaging MRI interval, per day	82	23	1.00	1.00–1.01	0.531
Main MRI to pathology interval, per day	82	23	1.00	0.99–1.02	0.720

Supplementary Table S8. Univariable logistic regression for T misclassification. Odds ratios were obtained from univariable logistic regression models using dichotomous T misclassification as the dependent variable. In the pooled analyses, the main MRI corresponded to staging MRI in the non-NAT cohort and restaging MRI in the NAT cohort. For tumor location/extent, the reference category was upper location. For cohort, the reference category was non-NAT. NE, not estimable or not reliably interpretable because of sparse data or quasi-complete separation leading to unstable or non-finite confidence intervals. Interval variables were only applicable to the NAT cohort.
