# Supplementary material for: Diagnostic Performance and Misclassification Patterns of Preoperative MRI in Rectal Cancer: A Real-World Study
Source: Diagnostics (Basel). 2026 May 13;16(10):1481. doi: 10.3390/diagnostics16101481 (PMC13205548; doi:10.3390/diagnostics16101481)
Supplement: Supplementary file 1 [file diagnostics-16-01481-s001.zip › Supplementary Table S9.pdf]

**Overall cohort**					
**Predictor**	**n**	**Events**	**OR**	**95% CI**	**P value**
Male sex	152	41	1.02	0.49–2.17	0.968
Age, per year	152	41	1.02	0.99–1.06	0.245
NAT cohort (vs non-NAT)	152	41	0.66	0.32–1.35	0.254
Tumor location/extent: lower (vs upper)	152	41	0.49	0.02–4.12	0.551
Tumor location/extent: lower + mid (vs upper)	152	41	0.30	0.08–0.90	0.046
Tumor location/extent: mid (vs upper)	152	41	0.31	0.10–0.81	0.024
Tumor location/extent: mid + upper (vs upper)	152	41	0.41	0.15–1.08	0.081
Mucinous component on baseline MRI	152	41	0.76	0.11–3.32	0.741
Predominantly mucinous appearance on baseline MRI	152	41	0.47	0.07–1.84	0.335
Tumor thickness on baseline MRI, per mm	152	41	1.01	0.97–1.05	0.642
MRF positive on baseline MRI	152	41	0.88	0.32–2.18	0.795
EMVI on baseline MRI	152	41	1.28	0.51–3.03	0.584
EMVI extension on baseline MRI, per mm	29	9	0.68	0.33–1.20	0.238
Tumor deposits on baseline MRI	152	41	0.53	0.03–3.42	0.568
Peritoneal reflection invasion on baseline MRI	152	41	3.60	1.21–11.01	0.021
Metastatic disease on baseline MRI	152	41	0.98	0.26–3.08	0.977
Main MRI to pathology interval, per day	152	41	1.01	0.99-1.02	0.381
**non-NAT cohort**					
**Predictor**	**n**	**Events**	**OR**	**95% CI**	**P value**
Male sex	70	22	1.29	0.45–3.92	0.645
Age, per year	70	22	0.99	0.93–1.04	0.606
Tumor location/extent: lower (vs upper)	70	22	1.57	0.06–41.95	0.756
Tumor location/extent: lower + mid (vs upper)	70	22	0.52	0.02–4.57	0.591
Tumor location/extent: mid (vs upper)	70	22	0.45	0.11–1.55	0.226

Tumor location/extent: mid + upper (vs upper)	70	22	0.39	0.05–1.85	0.278
Mucinous component on baseline MRI	70	22	NE	NE	NE
Predominantly mucinous appearance on baseline MRI	70	22	NE	NE	NE
Tumor thickness on baseline MRI, per mm	70	22	1.08	0.98–1.20	0.113
MRF positive on baseline MRI	70	22	NE	NE	NE
EMVI on baseline MRI	70	22	1.50	0.19–9.74	0.670
EMVI extension on baseline MRI, per mm	5	2	0.47	0.01–3.70	0.539
Tumor deposits on baseline MRI	70	22	NE	NE	NE
Peritoneal reflection invasion on baseline MRI	70	22	7.42	0.89–155.30	0.091
Metastatic disease on baseline MRI	70	22	NE	NE	NE
Main MRI to pathology interval, per day	70	22	1.00	0.97-1.03	0.890
**NAT cohort**					
**Predictor**	**n**	**Events**	**OR**	**95% CI**	**P value**
Male sex	82	19	0.79	0.28–2.31	0.660
Age, per year	82	19	1.05	1.00–1.11	0.085
Tumor location/extent: lower (vs upper)	82	19	NE	NE	NE
Tumor location/extent: lower + mid (vs upper)	82	19	0.24	0.04–0.98	0.059
Tumor location/extent: mid (vs upper)	82	19	0.18	0.02–0.85	0.048
Tumor location/extent: mid + upper (vs upper)	82	19	0.39	0.10–1.43	0.164
Mucinous component on baseline MRI	82	19	1.36	0.18–6.98	0.724
Predominantly mucinous appearance on baseline MRI	82	19	0.71	0.10–3.08	0.675
Tumor thickness on baseline MRI, per mm	82	19	1.00	0.95–1.05	0.872
MRF positive on baseline MRI	82	19	0.92	0.29–2.70	0.887
EMVI on baseline MRI	82	19	1.58	0.51–4.63	0.410
EMVI extension on baseline MRI, per mm	24	7	0.72	0.33–1.30	0.326
Tumor deposits on baseline MRI	82	19	0.64	0.03–4.35	0.697
Peritoneal reflection invasion on baseline MRI	82	19	3.39	0.87–12.93	0.070

Metastatic disease on baseline MRI	82	19	1.60	0.39–5.69	0.482
Baseline MRI to treatment interval, per day	82	19	1.00	0.99–1.00	0.308
Treatment to restaging MRI interval, per day	82	19	0.98	0.95–1.01	0.212
Baseline MRI to restaging MRI interval, per day	82	19	0.99	0.98–1.00	0.200
Main MRI to pathology interval, per day	82	19	1.01	0.99–1.02	0.345

Supplementary Table S9. Univariable logistic regression for N misclassification. Odds ratios were obtained from univariable logistic regression models using dichotomous N misclassification as the dependent variable. In the pooled analyses, the main MRI corresponded to staging MRI in the non-NAT cohort and restaging MRI in the NAT cohort. For tumor location/extent, the reference category was upper location. For cohort, the reference category was non-NAT. NE, not estimable or not reliably interpretable because of sparse data or quasi-complete separation leading to unstable or non-finite confidence intervals. Interval variables were only applicable to the NAT cohort.
