# Supplementary material for: Diagnostic Performance and Misclassification Patterns of Preoperative MRI in Rectal Cancer: A Real-World Study
Source: Diagnostics (Basel). 2026 May 13;16(10):1481. doi: 10.3390/diagnostics16101481 (PMC13205548; doi:10.3390/diagnostics16101481)
Supplement: Supplementary file 1 [file diagnostics-16-01481-s001.zip › Supplementary Table S10.pdf]

Non-NAT cohort, N misclassification - Predictor	OR	95% CI	P value
Peritoneal reflection invasion on baseline MRI	5.01	0.75–54.60	0.096
Tumor thickness on baseline MRI, per mm	1.07	0.98–1.18	0.144
**NAT cohort, T misclassification - Predictor**	**OR**	**95% CI**	**P value**
Age, per year	0.96	0.91–1.00	0.070
Metastatic disease on baseline MRI	2.58	0.76–8.67	0.125
**NAT cohort, N misclassification - Predictor**	**OR**	**95% CI**	**P value**
Peritoneal reflection invasion on baseline MRI	3.40	0.91–12.69	0.069
Age, per year	1.05	0.99–1.11	0.082

Supplementary Table S10. Cohort-specific multivariable models. Odds ratios were obtained from cohort-specific multivariable Firth logistic regression models using dichotomous MRI misclassification as the dependent variable. Final predictors were selected according to the parsimonious multivariable strategy described in the Statistical Analysis section. Note that no stable final multivariable model could be derived for T misclassification in the non-NAT cohort because of sparse data. In the NAT cohort, the main MRI corresponded to restaging MRI.
